# Career perspective: Jerome A. Dempsey

**DOI:** 10.1186/2046-7648-3-13

**Published:** 2014-07-07

**Authors:** Jerome A Dempsey

**Affiliations:** 1 John Rankin Laboratory of Pulmonary Medicine, University of Wisconsin-Madison, 1300 University Ave., Room 4245 MSC, Madison, WI 53706-1532, USA

**Keywords:** Respiratory physiology, Sleep, Mentoring, Hypoxia, Exercise

## Abstract

I received most of my education in Canada, finishing at the University of Wisconsin (UW)-Madison medical school, where I have remained throughout my academic career. The research in our laboratory centered on the broad field of respiratory and cardiorespiratory physiology and pathophysiology as applied to exercise, sleep, hypoxia, and several chronic disease states. We used a team approach to our research with much emphasis given to the training of basic and clinical scientists in respiratory physiology and medicine. Our trainees provide the most important and lasting legacy to our laboratory's efforts.

## 

I was born and grew up in London, Ontario, Canada, mentored by my mother and older brother James. Throughout high school, I had a strong interest in biology, math, and history and was taught by excellent teachers—but my life centered on team sports and my ambition was to be a professional baseball pitcher. Lack of the appropriate talent ended that dream by age 19 and my obtaining a Bachelor of Science degree from the University of Western Ontario allowed me to enter the teaching and coaching profession at the high school level in Hamilton, Ontario. At Western, I had been introduced to scientific inquiry by the enthusiastic example set by one of my professors, Michael Yuhasz. After 1 year of high school teaching, I attended the University of Alberta to study exercise physiology and obtained a MS degree, with mentoring provided by Max Howell and by a respiratory physician Brian Sproule—who had collaborated with Jere Mitchell on landmark studies in exercise physiology in Dallas in the late 1950s. My early research centered on the biological consequences of obesity and treatment interventions, and although these early studies were purely observational, I found my enthusiasm for research to be accelerating rapidly. For reasons which are now rather vague to me, my next step was to the University of Wisconsin-Madison graduate school—a wonderful institution of learning filled with endless seminars and with brilliant scientists whose doors were always open to curious students. After a year of dabbling in fat cell research, I met Dr. John Rankin (1923–1981) [1]^a^, a native of Glasgow, Scotland, professor of medicine and pulmonary physician at the University of Wisconsin (UW) who opened my eyes to the fascinating world of the respiratory system. By the mid-1960s, respiratory physiology was pretty well specialized into three areas, namely, lung and chest wall mechanics, gas exchange, and control of breathing. I was and still am intensely interested in addressing broad questions contained in the physiology of exercise, environmental hypoxia, sleep, obesity, and chronic obstructive pulmonary disease (COPD)—so our laboratory incorporated all three areas of respiratory physiology and eventually cardiorespiratory physiology into our studies of these topics. From the beginning, conducting studies of these broad topics became a team effort together with trainees and faculty. This team approach turned out to be extremely satisfying throughout my career, providing the opportunity to teach, conduct research, and to mentor with the likes of Bill Reddan, Peter Hanson, Lou Chosy, Bill doPico, Burt Olson, Jim Skatrud, Safwan Badr, Jerry Bisgard, Bert Forster, Phil Gollnick, Curt Smith, Les Proctor, Barb Morgan, and many more (see Figures 1 and 2). Dr. Rankin's capabilities, first as mentor and then as our department chairman, were truly extraordinary and were largely responsible for my remaining at the UW-Madison for my entire career (1968 to present) with wonderful sabbatical years to gain new skills at Sahlgrenska University in Sweden (for lung mechanics) and McGill University in Montréal (for respiratory muscle function). For most of my career, our laboratory used both chronically instrumented, unanesthetized animal models as well as humans and both healthy and disease models—to address different aspects of the same broad questions. This flexibility has been invaluable in allowing a more mechanistic approach in animals to help explain phenomena observed in the human. This approach also allowed some of our more extraordinary trainees to learn experimental approaches in both animals and humans.

**Figure 1:**
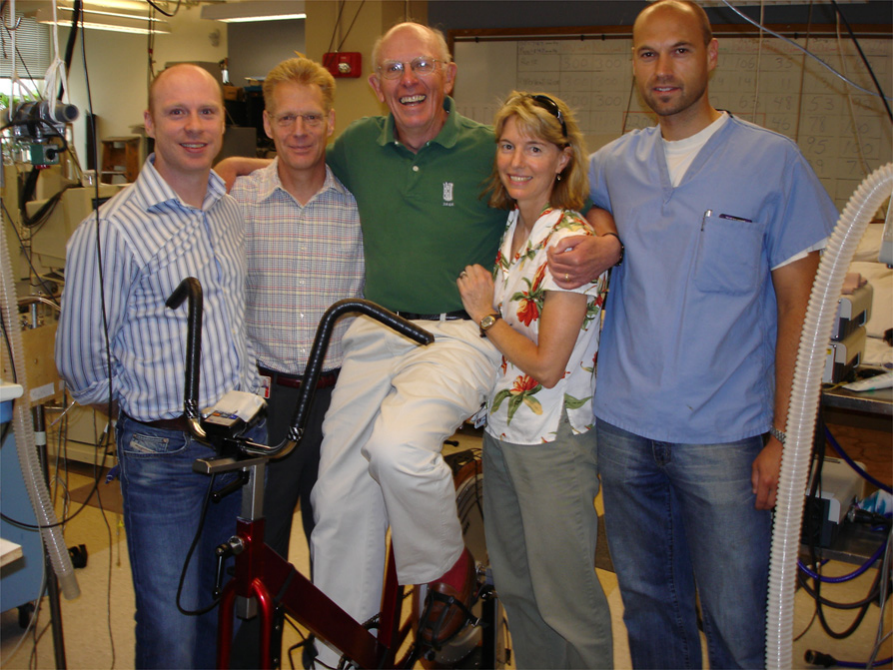
**Members and friends of the John Rankin Laboratory of Pulmonary Medicine, circa 2006.** Left to right, Lee Romer (post-doc), Marlowe Eldridge (faculty), Jerome Dempsey, Margaret Rankin (lab founder's daughter), and Markus Amann (post-doc).

**Figure 2:**
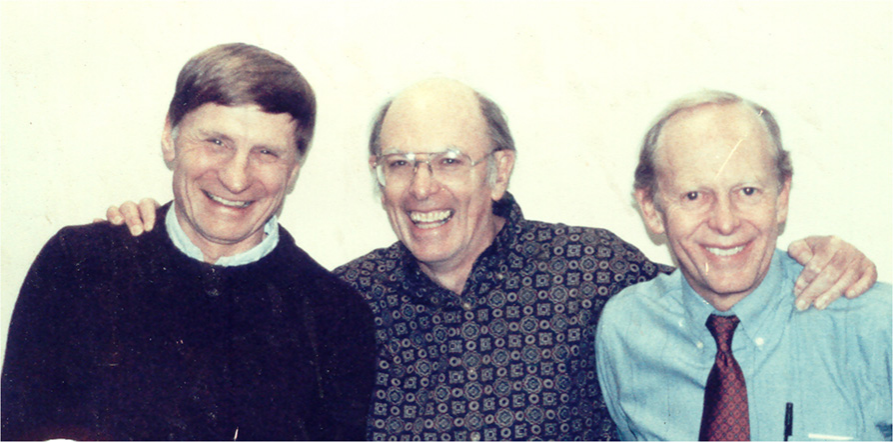
**The Wisconsin trio, Bert Forster, Jerry Dempsey, and Gerry Bisgard.** Three plus decades of exciting, fun-filled collaboration.

Important contributions of our laboratory over about a 45-year period may be grouped into five problem areas, each one encompassing a series of 10 to 25 studies conducted over several years.

• Hypoxic acclimatization research conducted in the mountains of Colorado and in hypobaric chambers provided evidence in several species which challenged a popular prevailing theory of the day to explain the role of brain fluid pH and chemoreceptor interactions in the regulation of breathing. These studies eventually led to the uncovering of a pivotal role for time-dependent sensitization of carotid chemoreceptors in hypoxia [2,3]. This decade-long debate impressed on me the vital importance of questioning prevailing theories - even those that are teleologically attractive and apparently ‘entrenched’ - and also the key role played by carefully obtained ‘negative findings’ in moving forward a field of inquiry. These studies also introduced me to how exciting and fruitful teamwork could be. Outside mentoring from such superb scientists as Vladimir Fencil of Harvard was invaluable during this early period, and Tom Hornbein provided an exemplary model of integrity and humility in science.

• The powerful role of CO_2_ chemosensitivity as a dominant regulator of breathing and breathing stability in sleep and as a key contributor to both central forms of apnea and even to the pathogenesis and treatment of obstructive sleep apnea [4,5]. Our research using sleeping humans also led to mechanical ventilation studies which revealed a powerful role for nonchemical, mechanical influences on ventilatory control, i.e., so called neuro-mechanical inhibition and its strong inhibitory ‘after effects’ after the mechanical ventilation was withdrawn [6,7]. Jim Skatrud and I introduced epidemiologists Terry Young and Mari Palta to the sleep and breathing field. This union of physiologists with population health scientists led to the birth of the Wisconsin Sleep Cohort (1988 to present) which, among other notable findings, was the first to report the prevalence of sleep disordered breathing in the general population [8] and also the consequences of sleep apnea to hypertension and mortality [9,10].

• Role of the ‘respiratory system’—gas exchange, airway mechanics, and respiratory muscle work and its cardiovascular interactions as significant contributors to O_2_ transport and exercise performance limitations in healthy endurance athletes as well as in COPD, chronic heart failure (CHF), and asthma [11,12].

• Contribution of cardiorespiratory interactions - both mechanical and reflex - in determining sympathetic vasoconstrictor outflow, stroke volume, and blood flow distribution during exercise in health and disease. A series of studies in animals and humans - both healthy and with CHF and COPD - established the importance of three types of reflex effects on sympathetic vasoconstriction during exercise, namely, (a) respiratory muscle metaboreflex, (b) carotid chemoreflex, and (c) type III to IV afferents from limb muscle [13,14]. Through our overall aim of organ system integration - along with a large measure of serendipity - studies of these reflex effects also led to the still controversial hypothesis that locomotor muscle fatigue was a carefully regulated variable during exercise with afferent feedback from fatiguing muscle providing an important influence over central output to locomotor muscle, i.e., so-called central fatigue.

• The use of chronically instrumented canines with separate perfusion of the isolated carotid chemoreflex provided evidence for (a) substantial contributions of carotid chemoreceptors to normal eupneic breathing, (b) stimulatory effects of global central nervous system hypoxia to ventilation, and (c) an interdependence of central chemoreceptor chemoresponsiveness to CO_2_ on the level of carotid chemoreceptor sensory input [15,16].

What do I think are the important questions that need to be addressed currently and in the future? Certainly, the mechanisms and sites of action through which respiratory CO_2_ exchange (VCO_2_) modulates ventilation needs to resurface and to be pursued because it represents the ‘underpinning’ of the control of breathing. Sufficient research has been completed on synaptic plasticity within the respiratory control system to reveal its vital importance in health and disease—but the underlying mechanisms and their pathophysiologic significance deserve more emphasis.

Conceptually, it is crucial that molecular/genetic methods be combined with traditional physiologic approaches and that future ‘physiologists’ be trained in both approaches. Federal funding of basic medical science and its associated training in the US is in disarray!…without this, as a priority, we are on the brink of losing a generation (or more) of scientists.

Where did the research questions and experimental approaches we used originate? I would like to think that these were our original ideas, but realistically, I know that interactions with countless colleagues and students and the literature - including many grant applications of others - provided the impetus for many of these studies. This long list of influences includes those scientists with whom we have been engaged significant controversies such as John Severinghaus, Brian Whipp, Tom Hornbein, Fred Eldridge, and Magdy Younes. Controversy breeds progress!

It is important to point out that these areas of discovery outlined above are evolving and have and will most certainly continue to undergo major revisions over time. Clearly, the most lasting legacy of the John Rankin Laboratory and my involvement is its 68 pre- and postdoctoral and 90–100 undergraduate trainees—including physiologists, veterinary scientists, biomedical engineers, and pulmonary and sleep physicians. It took me a very long time and in the wake of many errors to learn how to mentor according to the example set by my mentor John Rankin who emphasized the importance of recognizing the potential for growth and success in our trainees - regardless of background - and to nurture this potential with continuous positive reinforcement. Instilling an enthusiasm for scientific inquiry is all important! My career in science and teaching has provided me and my family more than anyone could wish for. My only regret is that I did not learn and practice some of these important mentoring attributes a touch earlier. Most assuredly, the Rankin lab alumni family is my most significant and rewarding achievement and of the several ‘old guy accolades’ awarded me in recent years I most cherished last year's APS Bodil Schmidt Nielsen award because it recognized both the mentoring and science provided over 45 years by the Rankin lab team of mentors and trainees [17] (see Figure 3). It has been a great ride!

**Figure 3:**
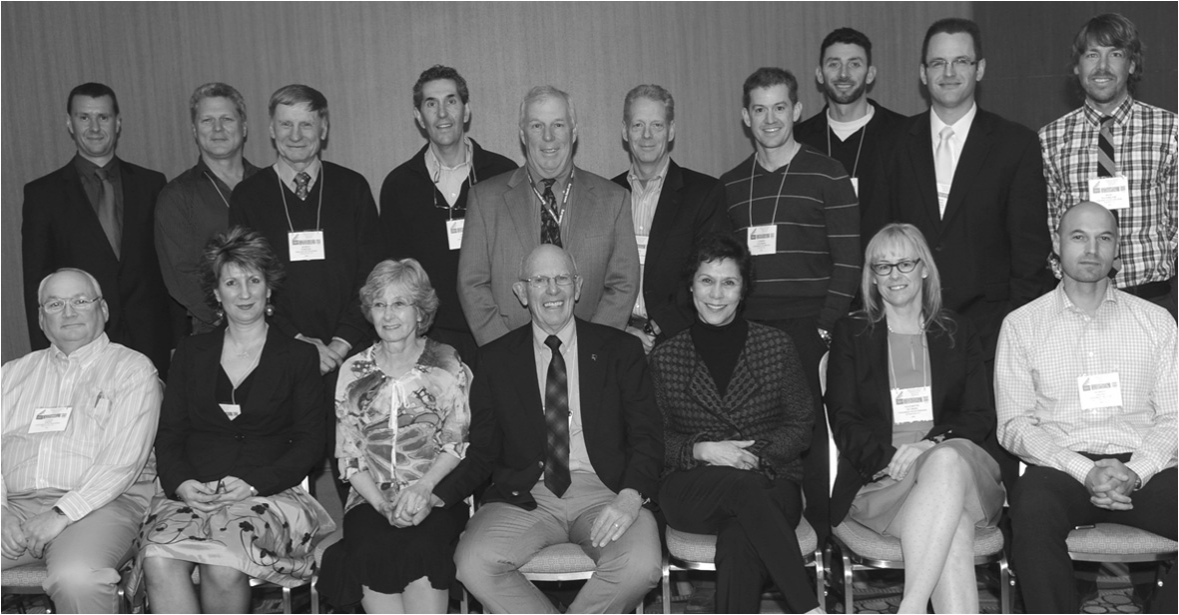
**UW-Rankin Laboratory personnel and alumni attending the Bodil Schmidt-Nielsen Award Ceremony at Experimental Biology, 2013.** Top row, left to right, Bruno Chenuel, Gordon Mitchell, Bert Forster, Ralph Fregosi, Tim Musch, Marlowe Eldridge, Andy Lovering, Noah Marcus, Jordan Miller. Bottom row, Curt Smith, Mihaela Teodorescu, Barb Morgan, Jerry Dempsey, Anne Berssenbrugge, Claudette St. Croix. Reproduced with permission from [17].

## Consent

Written informed consent was obtained from the participants for publication of the accompanying images in this manuscript. The consent form is held by the author and is available for review by the Editor-in-Chief.

## Endnotes

^a^Inventor of the Glasgow scale or Rankin scale in the 1950s for prognosis of stroke patients—still used today in population studies [1].

## Competing interests

The author declares that he has no competing interests.
